# The Efficacy of Individualized Music Therapy in Patients With MCS: A Pilot Study on EEG

**DOI:** 10.1155/np/8832006

**Published:** 2026-07-10

**Authors:** Xiaoying Zhang, Yunlei Wang, Zuliyaer Talifu, Qingqing Feng, Jiayi Gu, Xiaobing Li, Xiaoxia Du

**Affiliations:** ^1^ School of Rehabilitation Medicine, Capital Medical University, Beijing, China, ccmu.edu.cn; ^2^ Music Therapy Center, China Rehabilitation Research Center, Beijing, China, crrc.com.cn; ^3^ Department of Neurorehabilitation, China Rehabilitation Research Center, Beijing, China, crrc.com.cn; ^4^ School of Population Medicine and Public Health, Chinese Academy of Medical Sciences/Peking Union Medical College, Beijing, China, pumc.edu.cn; ^5^ Department of Rehabilitation, Hunan Provincial People’s Hospital, The First Affiliated Hospital of Hunan Normal University, Changsha, China, hunnu.edu.cn; ^6^ Department of Music Artificial Intelligence and Music Information Technology, Central Conservatory of Music, Beijing, China

**Keywords:** disorders of consciousness (DoC), electroencephalography (EEG), individually music therapy (MT), MATADOC (Music Therapy Assessment Tool for Awareness in Disorders of Consciousness), minimally consciousness state (MCS)

## Abstract

**Objective:**

To evaluate the feasibility, safety, and preliminary effects of individual music therapy (IMT) on electroencephalography (EEG) cortical activation in patients with minimally conscious state (MCS).

**Design:**

This study was pilot randomized, single‐blind, and blank‐controlled trial.

**Setting:**

China Rehabilitation Research Center (CRRC), Beijing, China.

**Participants:**

Sixty‐two MCS patients (diagnosed by Coma Recovery Scale‐Revised [CRS‐R]) were enrolled between March 2024 and July 2025; 43 completed the trial and were randomized.

**Intervention:**

The experimental group (*n* = 22) received 12‐week individualized music therapy (30‐min sessions, five times per week) delivered by a certified music therapist.

**Comparator:**

The control group (*n* = 21) received routine treatment and rehabilitation training without music intervention.

**Primary outcome:**

The change in EEG spectral power (alpha band, 8–13 Hz) over right‐hemisphere frontocentral regions from baseline (T1) to post‐intervention (T2).

**Results:**

The IMT group showed significantly increased alpha power over the right lateral prefrontal cortex, precentral gyrus, and central sulcus at T2 compared T1 (*p* < 0.05; descriptive comparison), whereas no such change was observed in the control group. No adverse events occurred in either group.

**Conclusion:**

IMT is feasible and safe in medically fragile MCS patients and may induce right‐hemisphere cortical activation. These pilot findings are hypothesis‐generating and require confirmation in larger, adequately powered trials.

**Trial Registration:**

Chinese Clinical Trial Registry: ChiCTR2500114153, MR‐11‐24‐014167

## 1. Introduction

The minimally conscious state (MCS) is a distinct condition within the spectrum of disorders of consciousness (DoC). MCS is characterized by weak but discernible behavioral responses, indicating partially preserved cognitive processing abilities. Electroencephalography (EEG)‐based studies have demonstrated that music therapy elicits unique electrophysiological markers associated with improved consciousness in patients with MCS. Using scalp EEG recordings, Alnagger et al. [1] found that patients with MCS exhibited significantly greater neural oscillation consistency at the frequency of musical beats than those in the vegetative state/unresponsive wakefulness syndrome (VS/UWS) [2]. This synchronization reflects the enhanced coupling between auditory stimuli and neural oscillations [3]. Notably, theta‐gamma phase–amplitude coupling (PAC) is markedly strengthened during exposure to personalized music, and this enhancement positively correlates with improvements in clinical behavioral scores [4]. The cross‐frequency neural coordination index reflects the efficiency of information transfer across different scales of the neuronal population. Its augmentation suggests that music may facilitate the recovery of consciousness by enhancing the integrative functions of the thalamocortical circuits and cortico‐subcortical networks [5].

Neuroimaging studies have confirmed that personalized music can significantly enhance functional connectivity between the default mode network (DMN) and the dorsal attention network. These constitute a fundamental neural substrate for maintaining self‐awareness and environmental awareness [6]. Importantly, evidence has shown that the enhancement of *θ*‐γ PAC serves as a key electrophysiological indicator of music‐induced improvements in consciousness. This effect is particularly pronounced in patients in the MCS and is positively correlated with clinical behavioral scores [7]. Previous studies have also indicated that individual music therapy (IMT) promotes motivation formation through emotional arousal in patients with MCS, thereby facilitating the limbic system activation. Strong rhythmic stimulation engages the brainstem–thalamus–cortex pathway to reconstruct movement timing, alleviate deficits in movement initiation, and enable auditory‐motor integration [8]. Furthermore, EEG investigations have found that following music stimulation, the amplitude of mismatch negativity (MMN) increases, reflecting attention processing capacity recovery.

The efficacy of music therapy in patients with MCS critically depends on the individualized adaptation of stimulation parameters. This optimization process requires the integration of multidimensional information, including neurophysiological profiles, etiological factors, and behavioral responses. Tempo, a core parameter in the temporal domain, influences the level of consciousness by modulating the synchrony of neural oscillations [9]. Research has shown that different tempos elicit distinct EEG power spectrum responses. For example, slow tempos (56 bpm) significantly increase frontal theta wave (4–8 Hz) and alpha wave (8–12 Hz) power, corresponding to a rest‐relaxation state, while fast tempos (156 bpm) enhance beta (12–35 Hz) and gamma (>35 Hz) wave activity, indicating increased recruitment of cognitive resources [10]. Notably, tempo represents only one dimension of the musical expression. Relying solely on tempo is insufficient to capture the nuanced characteristics of therapeutic interventions in IMT. Melody, a significant form of cognitive and emotional information, plays a more effective role in inducing brain‐sound synchronization via the emotional network, surpassing the arousal‐promoting effects of tempo alone [11].

The application of music therapy to MCS has evolved from single‐modality interventions to multimodal approaches, generating synergistic effects through integration with other neuromodulation techniques [12]. How do music‐induced oscillations propagate more effectively across synapses? How does the temporal structure of music provide rhythmic pacing for neural cluster discharges, thereby forming an “excitatory‐rhythmic synergy” that supports patient awakening? In conjunction with the Music Therapy Assessment Tool for Awareness in DoC (MATADOC) framework, this study employed high‐channel electroencephalogram monitoring technology to deliver innovative IMT to patients with MCS [13]. Each patient’s IMT plan was customized, integrating rhythm and melody classifications with individually relevant autobiographical memories. This study aimed to evaluate the behavioral impact of IMT in promoting patient emergence from MCS and to explore its relationship with the underlying brain network dynamics [14].

## 2. Subjects and Methods

### 2.1. Trial Registration and Ethical Approval

This study was approved by the Ethics Committee of the China Rehabilitation Research Center (CRRC) on March 19, 2024 (Approval Number 2024‐018‐2). Informed consent was obtained from all participants or their legally authorized representatives prior to enrollment. The trial was prospectively registered in the China National Medical Research Registration and Filing Information System (Primary Registry). All primary and secondary outcomes, as well as the analysis plan, were prespecified in the protocol submitted to the primary registry.. The content of this registration is identical to that of the primary prospective registration, and no protocol changes were introduced. The final manuscript has been verified against the primary registered protocol, and no deviations exist.

### 2.2. Participants

This study enrolled 62 patients from the CRRC between March 2024 and July 2025. Ultimately, 43 patients completed the trial, while 19 patients dropped out. The inclusion and exclusion criteria are as follows:

Inclusion criteria: (1) diagnosis of MCS confirmed using the Coma Recovery Scale‐Revised (CRS‐R) and the Glasgow Outcome Scale‐Extended (GOS‐E), score ≥8 for auditory or motor subscales, with evidence of reproducible command following or purposeful behavior [15, 16]; (2) inpatient status with a minimum illness duration ≥1 months (range: 1–12 months); (3) adult aged between 18–70 years; (4) ability to remain in a supine position and tolerate the intervention for at least 30 min; (5) no prior formal music education; and (6) written informed consent obtained from both the patient and their family members.

Exclusion criteria were as follows: (1) presence of severe or malignant arrhythmia or a history of cardiac surgery; (2) orthostatic hypotension; and (3) significant hearing loss. The criteria for withdrawal or termination included changes in the patient’s condition, discharge, or voluntary withdrawal from the study, which allowed for timely discontinuation of the intervention The participant characteristics are summarized in Table 1.

**Table 1:** Baseline participant characteristics.

Category	Intervention group (mean ± SD)	Control group (mean ± SD)	*p*‐Value
Minimally consciousness state (MCS)	22	21	>0.05
Gender	—	—	0.172
Male	15	10	—
Female	7	11	—
Pathogeny	—	—	>0.05
Hemorrhagic stroke	14	12	—
Trauma brain injury	5	6	—
Hypoxic‐ischemic	1	1	—
Ischemic stroke	1	1	—
Subarachnoid hemorrhage	0	1	—
Encephalitis	1	0	—
Age	53.82 ± 16.08	52.35 ± 19.03	0.700
Time since injury (month)	2.07 ± 1.76	2.30 ± 1.52	0.559
Education background	—	—	>0.05
Primary school	10	10	—
Junior high school	9	10	—
Bachelor degree or above	3	1	—

*Note*: Table 1 summarizes the demographic and clinical characteristics of the two patient groups, including gender distribution, age, duration of injury, and educational background. Continuous variables are presented as mean ± standard deviation. The intervention group received individual music therapy. The blank control group received standard medical care. A *p*‐value greater than 0.05 suggests no statistically significant differences among the groups.

The 43 patients were randomly assigned to one of two groups. Those in the intervention group received IMT (*n* = 22). Those in the blank control group received routine medical care (*n* = 21). The two groups received identical routine treatments and rehabilitation training. No statistically significant differences were observed between the groups in terms of sex distribution, age, time since injury, or educational background (*p*  > 0.05).

### 2.3. Study Design

This 2‐group clinical investigation adopted a randomized controlled trial (RCT) framework structured using a pretest–posttest approach. In this study, a randomized single‐blind design was adopted to control bias. Given that patients in the MCS are unable to comprehend or respond to group allocation information, the “single‐blind” arrangement mainly refers to the masking of group information from outcome assessors and data analysts. Specifically, operators responsible for EEG data acquisition, preprocessing, and feature extraction were unaware of whether patients were assigned to the personalized music therapy group or the control group. Music therapists provided individualized interventions under an open‐label scheme. All EEG data were anonymized prior to analysis, and unblinding was not performed until the completion of the statistical analysis. Participants and their families were informed of the general nature of the clinical study only after providing written informed consent. Masking procedures were performed during group allocation and data analysis to ensure objectivity. Participants in the intervention group received IMT, while those in the control group received routine treatment and rehabilitation training, except for music therapy.

### 2.4. Procedure

Following approval from the CRRC Ethics Committee and official registration of the clinical trial, initial participant screening was conducted by neurosurgeons. Patients who were preliminarily classified as being in a MCS according to the MATADOC were then referred to the music therapy department for further evaluation. Music therapy specialists reviewed the inclusion and exclusion criteria to identify eligible participants. Once eligibility was confirmed, the researchers contacted the patient’s family members, provided detailed information about the study, and obtained written informed consent. This information covered the study’s objectives, procedures, potential risks and benefits, confidentiality measures, and participants’ rights.

**Figure 1:**
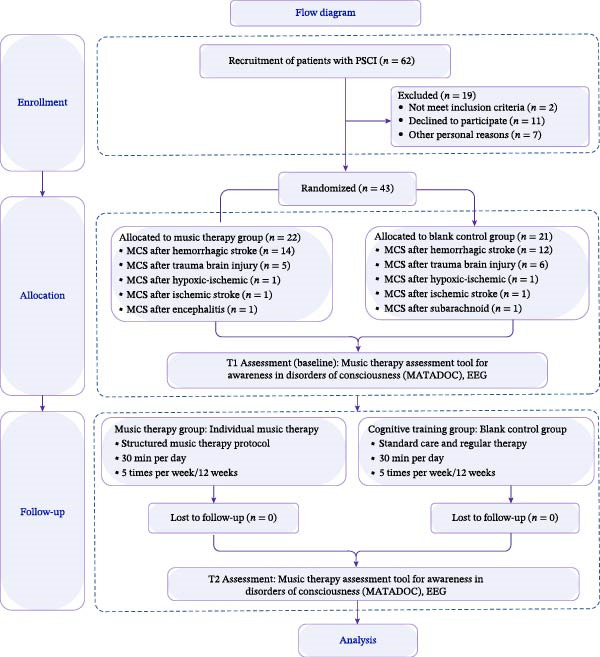
Participant flow diagram. CONSORT flowchart for enrollment, allocation, and completion of study participants. A total of 43 participants were enrolled in the study, and all individuals completed the full course of the trial. The intervention group (*n* = 22) received personalized music therapy administered by a certified music therapist, whereas the blank control group (*n* = 21) received no additional interventions beyond routine treatment and physical therapy. Two assessment points were scheduled throughout the study period: T1 (baseline measurement) and T2 (post‐intervention assessment). The final data analyze was based on a cohort of 43 patients diagnosed with a minimally conscious state.

After enrollment, participants’ MATADOC scores were reviewed to assess for any hearing feedback. Subsequently, a computer‐generated randomization sequence (created using Excel 2013, Microsoft Office, Seattle, WA, USA) was used to assign patients to one of two groups. The intervention group received 12 weeks of individual music therapy sessions conducted by a certified music therapist. The control group received no additional interventions beyond routine treatment and physical therapy. The flow of participant enrollment and group allocation is illustrated in Figure 1.

### 2.5. Interventions

The treatment intervention was initiated following enrollment. All patients in the intervention group received IMT from one of the certified and licensed music therapists, while those in the control group received no additional interventions beyond routine treatment and physical therapy. Other than IMT, rehabilitation treatment methods were the same in both groups. For the IMT group, each patient underwent 30 min of intervention 5 days per week for 12 weeks. For the blank control group, routine support was provided by registered physicians, nurses, and therapists.

The individualized music therapy was provided by a certified music therapist and monitored by the neurologist.

#### 2.5.1. Intervention Preparation

The IMT sessions were conducted in a private single‐patient treatment room. Throughout therapy, the environment was maintained in a quiet state, free from external sounds and electromagnetic interference, except for those directly associated with music therapy, to minimize noise interference. Concurrently, EEG data were collected using a 128‐channel high‐resolution EEG system. Prior to the experiment, the objectives, procedures, and relevant precautions were thoroughly explained to each patient’s family members to ensure comprehensive understanding and voluntary participation. Family members were also informed that, during event‐related potentials (ERPs) testing, the patient should remain in a stable physiological condition, avoid excessive fatigue or emotional arousal, and exhibit no significant coughing or expectoration.

#### 2.5.2. Task Paradigm

Based on the research objectives, a music therapy paradigm was designed and implemented. The treatment protocol integrated musical playback, singing, and rhythmic stimulation. Individualized music therapy, implemented by a music therapist, followed a standardized procedure consisting of a fixed sequence of songs being played and sung. Song selection was based on the patients’ personal and social life histories to structure the thematic sequence. The 30‐min structured music stimulation protocol is as follows: (1) The opening segment was the “Name Calling Song” (2.5 min), adapted from the Xinjiang Uyghur folk song “Mayila” for the purpose of calling patients’ names. (2) Treatment segment: The thematic content was primarily a curated set of songs selected according to the patients’ personal and social life histories (25 min). (3) The closing segment was the “Goodbye Song” (2.5 min), with lyrics composed by the therapist. For instance, one patient had participated in the Liberation War during her youth and therefore exhibited a clear preference for revolutionary songs. Accordingly, the patient’s personalized song set included many patriotic and military‐themed songs. For example, the patriotic song “Sing a Mountain Song to the Party” and the military‐themed song “I Love the Blue Sky of My Motherland,” among others, were selected to elicit strong emotional and behavioral responses. During the therapy session, when the therapist sang these songs in real‐time, the patient’s vital signs were simultaneously recorded and monitored. The implementation of music therapy is conducted by certified and registered music therapists, delivered to patients via one‐on‐one live singing, with concurrent 128‐channel electroencephalogram monitoring. Each session lasts 30 min, with five sessions per week, over a total duration of 12 consecutive weeks.

#### 2.5.3. Blank Control Group

For patients in the blank control group, standard care was delivered according to the institutional protocol for patients with DoC. The care was provided by the bedside nursing team under the supervision of a rehabilitation physician, with daily monitoring conducted by a neurologist. The intervention period lasted 12 weeks (identical to that in the music therapy group). During this period, each patient received the following routine care components: (1) twice‐daily nursing rounds (each lasting ~15 min) for safety protection, airway clearance, basic positioning, and prevention of secondary injuries; (2) continuous physiological monitoring (heart rate, blood pressure, oxygen saturation, and respiratory rate) recorded every 4 h; and (3) once‐weekly nutritional adjustment and etiological management (e.g., anti‐infection or antispasticity medications as prescribed). No music intervention, structured auditory stimulation, or extra social contact beyond standard care was provided to the control group. All monitoring and outcome assessments were performed at the same time points (baseline, day 7, and day 14) as in the music therapy group.

### 2.6. Measurements

Before the intervention, all participants were assessed at baseline by the researcher using (1) the MATADOC [17] for behavioral assessment and (2) electroencephalogram (EEG). Behavioral and EEG changes were also observed in week 12 (i.e., the second assessment).

#### 2.6.1. MATADOC

MATADOC is a behavioral assessment method that assesses the degree of consciousness of patients [18]. MATADOC is an auditory‐based assessment tool specifically designed for patients with DoC [19]. The assessment has demonstrated superior sensitivity in detecting covert signs of awareness in these patients. The scale comprises six subscales that assess the key behavioral domains relevant to the consciousness evaluation. MATADOC has been validated as an instrument with strong internal consistency, inter‐rater reliability, and structural validity. The assessment outcomes showed complete agreement (100% consistency) with the established external reference standards [20]. Taken together, MATADOC is a valuable behavioral assessment instrument that supports multidisciplinary evaluations of consciousness in individuals with DoC [17].

Briefly, MATADOC assesses patients with DoC across the following 15 dimensions: (1) sight, (2) hearing, (3) stimulus to musical sensation, (4) verbal command, (5) state of consciousness, (6) behavioral reaction to music, (7) reaction to music, (8) nonverbal communication, (9) decision‐making, (10) mobility, (11) attention to tasks, (12) directed actions, (13) emotional reactions, (14) vocalization, and (15) overall (total score). A previous study demonstrated that, after 12 weeks, the intervention group demonstrated statistically significant improvements compared with the control group in the stimulus to musical sensation, hearing, sight, vocalization, and total score [18]. The MATADOC was administered in its original English version by a certified music therapist who had completed the official MATADOC Level 1 training (Royal Hospital for Neuro‐disability, UK) in July 2018. No formal inter‐rater reliability was calculated in this pilot study as all assessments were performed by the same trained rater.

#### 2.6.2. EEG

An EEG is a record of the electric signal generated by the cooperative action of brain cells or, more precisely, the time course of the extracellular field potentials generated by their synchronous action [21]. EEG is indispensable for the diagnosis and management of patients with MCS. EEG is used to objectively quantify the functional status of the brain, predicting the likelihood of recovery, and guiding targeted interventions [22]. In the diagnosis and classification of DoC, EEG serves as a critical diagnostic tool for differentiating between VS/UWS, MCS, and MCS plus/minus (MCS^+/−^) based on characteristic changes in the patient’s brainwave patterns. A key distinction between MCS and VS is that patients with MCS may exhibit alpha rhythm reorganization (8–12 Hz) during eye closure and generate ERP in response to auditory stimuli, indicating the presence of residual cognitive processing [23].

Data segmentation was defined with the onset of music playback set as 0 ms, where the baseline power was measured from −1000 ms prior to the onset of music, and the task concluded at 2000 ms post‐onset. This approach enabled the observation of cerebral functional changes occurring within a 2‐s window during the music therapy session. The electrodes were positioned in accordance with the International 10–20 system. The electrodes were applied to the patient’s scalp using a saline‐based cap to ensure optimal contact, with the electrode impedance being maintained below a predefined threshold. The EEG recording system was activated prior to the commencement of the music therapy session for continuous monitoring of the patient’s cerebral electrical activity.

### 2.7. Outcome Measures

The primary outcome was the change from baseline in frontal α‐band (8–12 Hz) and γ‐band (40 Hz) derived from resting‐state EEG, measured at baseline and week 12 (post‐intervention) compared to day 0 (baseline). The rationale for selecting a neurophysiological endpoint as the primary outcome was twofold: (1) patients with MCS often exhibit fluctuating behavioral responses, making behavioral scales prone to ceiling/floor effects and observer bias and (2) EEG‐derived markers (particularly alpha power in frontoparietal regions) have been shown to correlate with residual consciousness levels and are less susceptible to motor or arousal confounds.

Secondary outcomes included change in the total score of the MATADOC from baseline to week 12. Subdomain scores (e.g., auditory function, visual pursuit, and motor response) were exploratory. Between‐group comparison of EEG power in α and γ bands at week 12. Within‐group change in the MATADOC total score from baseline to week 12. Correlation between change in EEG alpha power and change in MATADOC total score was observed at week 12. The primary analysis time point was week 12. All outcome assessments were conducted by blinded assessors. No multiplicity adjustment was applied for secondary outcomes, as this was a pilot study intended to generate hypotheses.

### 2.8. Statistical Analysis

#### 2.8.1. Scale Data Processing

Statistical analyses were performed using Stata (version 17.0) and SPSS (version 23.0). Descriptive statistics were presented as the mean ± standard deviation (SD). Demographic and clinical characteristics (age, duration of illness, and baseline MATADOC scores) were compared between the two groups (IMT vs. standard care) using independent sample *t*‐tests. Categorical variables (sex, diagnosis, and etiology) were analyzed using the Chi‐square test or Fisher’s exact test, as appropriate. To evaluate the therapeutic effect of IMT, a 2 × 2 mixed‐design ANOVA was employed with Group (IMT vs. control) as the between‐subjects factor and time (T0: baseline vs. T1: 12 weeks) as the within‐subjects factor. The primary focus was the group × time interaction effect, which indicates whether the recovery rate significantly differed between groups. Given the observed baseline differences in MATADOC total scores, an analysis of covariance (ANCOVA) was performed as a robust secondary analysis. The post‐intervention (T1) score was used as the dependent variable, the group as the fixed factor, and the baseline (T0) score as the covariate to isolate the net effect of the intervention. ANCOVA was applied to each category to assess group‐wise improvements. Exact *p*‐values were reported for all analyses, with a two‐tailed *p* < 0.05 considered statistically significant.

#### 2.8.2. EEG Data Processing

All EEG preprocessing and feature extraction procedures were conducted by analysts blinded to group allocation (music therapy vs. control). The group code was not disclosed until all primary and secondary analyses were completed. The EEG regions of interest (ROIs) and time–frequency windows of interest were prespecified prior to data analysis based on previous studies on auditory processing and consciousness recovery [24].

##### 2.8.2.1. Equipment and EEG Acquisition

EEG data were recorded using a NeuSen HEEG series digital electroencephalograph (China, Changzhou; Medical Device Registration No.: SuXieZhuZhun 20202071023) with a 128‐channel configuration. The sampling rate was 16,000 Hz for all channels, supporting a wide frequency band from 0 to 4 kHz. Electrodes were placed according to the international 10–20 extended system, covering the entire scalp. The electrode impedance was maintained below 5 kΩ. The reference electrode was CPz (double CPz) and the ground electrode was placed at AFz.

##### 2.8.2.2. EEG Preprocessing

Preprocessing was performed in MATLAB using the EEGLAB toolbox. After re‐referencing, bandpass filtering (0.5–50 Hz), and interpolation of bad channels, independent component analysis (ICA) was applied to decompose the continuous EEG data. Based on the scalp topography, temporal waveform, and power spectrum of each independent component, artifactual components corresponding to electrooculography (EOG), electromyography (EMG), and electrocardiography (ECG) were identified and removed. The remaining components were then reconstructed to obtain clean EEG signals for subsequent time–frequency analysis and ERP computation.

##### 2.8.2.3. Event‐Related Spectral Perturbation (ERSP) Analysis

After basic preprocessing (common average re‐referencing, ICA artifact removal, and bad channel interpolation), data were epoched from –400 to 1400 ms relative to the music stimulus onset, with the baseline period defined as –400 to 0 ms. ERSP (in dB) and inter‐trial coherence (ITC) were computed in the 5–50 Hz frequency range using wavelet transform with cycles = [3, 0.5]. Results are presented for electrode F6. The ERSP color scale was set to –4–4 dB and the ITC color scale to 0–1. The ERP waveform was overlaid on the ERSP and ITC plots.

##### 2.8.2.4. Cluster‐Based Permutation Test

For each electrode, independent *t*‐tests were performed, followed by false discovery rate (FDR) correction across all electrodes with a *q*‐value threshold of  ^∗^ *p* < 0.05, indicating a statistically significant difference.

##### 2.8.2.5. Superimposed Averaging

The EEG data corresponding to the musical pieces administered during the therapy session were superimposed and averaged for each subject to extract ERP components associated with specific auditory stimuli.

##### 2.8.2.6. Waveform Analysis

The latency, amplitude, morphology, and related parameters of the ERP waveforms were analyzed to elucidate the neural processing of auditory stimuli induced by music therapy.

**Figure 2:**
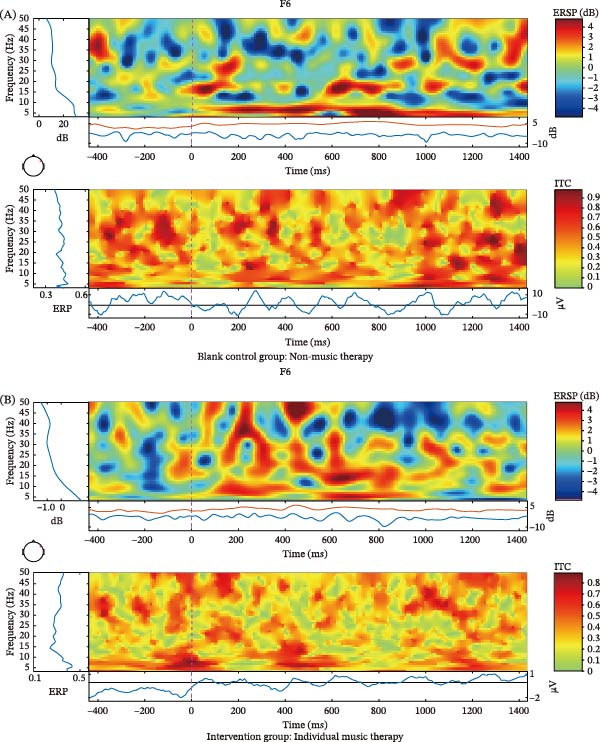
Time‐frequency analysis of event‐related spectral perturbation (ERSP) and inter‐trial phase locking (ITP) for the blank control group (A) and IMT group (B). Red indicates power increase relative to baseline (>0 dB); blue indicates power decrease (<0 dB). In the α‐band (8–12 Hz) at 200–400 ms, ITP exceeded 0.5, which is consistent with phase‐locked auditory processing reported in previous studies. In the γ‐band (40 Hz), ITP remained below 0.2, indicating nonphase‐locked activity. The time–frequency plane is presented for descriptive purposes; no statistical comparisons were performed across groups for each time–frequency pixel (software analysis by Neuracle‐Seal).

To account for multiple comparisons across multiple dimensions, the following approach was adopted: Across channels (topographic analysis): Cluster‐based permutation tests (5000 permutations) controlling FWER at  ^∗^ *p* < 0.05, indicating a statistically significant difference. Across time points in ERSP, for the prespecified ROIs, time–frequency windows of interest were defined a priori, minimizing the need for pointwise corrections. For exploratory visualization of ERSP topography across the full time–frequency plane (Figure 2), no inferential statistics were performed; Figure 2 is presented for descriptive purposes only. Across multiple secondary outcomes: given the exploratory nature of this pilot study (*N* = XX), secondary analyses were not corrected for multiple comparisons. Results from secondary outcomes should be interpreted as hypothesis‐generating, and confirmatory testing is required in future studies.

## 3. Results

### 3.1. Baseline Characteristics

A total of 43 participants (IMT group: *n* = 22 and control group: *n* = 21) were included in the final analysis. As shown in Table 1, there were no significant differences between the two groups in terms of age (*p* = 0.701), gender (*p* = 0.172), or months since injury (*p* = 0.559). However, the IMT group exhibited a significantly higher baseline MATADOC total score compared to the control group (13.36 ± 1.89 vs. 11.29 ± 2.00, *p* = 0.001). This baseline imbalance was statistically addressed in subsequent efficacy analyses using ANCOVA.

### 3.2. Primary Efficacy: MATADOC Total Score

**Figure 3:**
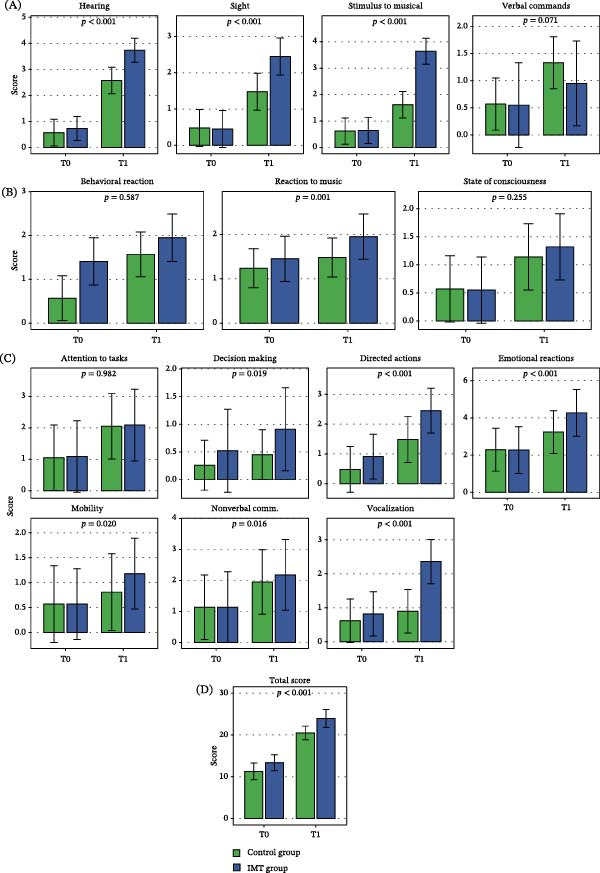
Comparison of results of MATADOC in the two groups. The 15 items were derived from the 15 dimensions of MATADOC.  ^∗∗^ *p*﹤0.01, remarkable significance;  ^∗^ *p*﹤0.05, significance. (A) Subgroup 1: scoring main fundamental ability scoring (Yes 2/No 1). (B) Subgroup 2: scoring: evaluation of the specific type of music used. (C) Clinical information used to establish targets and interventions. (D) MATADOC total score.

To evaluate the effect of IMT on behavioral responsiveness, a 2 × 2 mixed‐design ANOVA was conducted (as detailed in Table 2 and Figure 3). The analysis revealed a highly significant group × time interaction (*F* [1, 41] = 208.58, *p* < 0.001), indicating that the recovery trajectory of the IMT group was significantly superior to that of the control group. A significant main effect of time was also observed (*F* [1, 41] = 3569.88, *p* < 0.001), reflecting general recovery in both groups.

**Table 2:** Interaction and adjusted group effects for MATADOC scores.

Variable	Time	IMT group (*n* = 22)	Control group (*n* = 21)	Interaction (*F*)	Adjusted diff (Beta)	*p*‐Value
Total score	T0	13.36 ± 1.89	11.29 ± 2.00	208.58	−7.61	<0.001
	T1	23.95 ± 2.15	20.48 ± 1.63	—	—	—

Given the baseline imbalance, ANCOVA was performed with the T0 score as a covariate. The results confirmed that after adjusting for baseline scores, the IMT group still achieved significantly higher total scores at 12 weeks compared to the control group (adjusted difference = 7.61, *p*  < 0.001). The IMT group showed an average improvement of 10.59 points (from 13.36 to 23.95), while the control group improved by 9.19 points (from 11.29 to 20.48).

Subdimension analysis:

1. To further elucidate the intervention effect, MATADOC dimensions were grouped into three functional categories, and each sub‐item was analyzed (Table 2). Main fundamental ability (dimensions 1–5): This category assesses primary sensory and conscious responses. The IMT group showed significantly greater improvement (adjusted difference: −3.86, *p* < 0.001). Specifically (Table 3), hearing and stimulus to musical sensations demonstrated the most robust gains, with the IMT group consistently achieving higher scores at T1 (*p* < 0.001). Improvements in sight were also significantly greater in the IMT group (*p* < 0.001). While verbal commands and state of consciousness improved in both groups, the adjusted group differences did not reach statistical significance (*p* = 0.071 and *p* = 0.255, respectively).
2. Music specificity (Table 3, dimensions 6–7 and Figure 3): significant group differences were observed in responses specifically triggered by music (*p* = 0.036). Although behavioral reaction to music showed improvement in both cohorts without a significant adjusted group difference (*p* = 0.587), the specific reaction to music was significantly more advanced in the IMT group (*p* = 0.001).
3. Other clinical information (Table 3, dimensions 8–14 and Figure 3): the IMT group exhibited superior performance in clinical targets related to communication and motor output (adjusted difference: −3.82, *p* < 0.001). Key improvements were noted in vocalization (*p* < 0.001), directed actions (*p* < 0.001), and emotional reactions (*p* < 0.001), where the IMT group showed markedly higher T1 scores. Other dimensions like nonverbal communication, decision making, and mobility also showed significant advantages for the IMT group (*p* < 0.05), while attention to tasks showed equivalent improvement in both groups (*p* = 0.982).

#### 3.2.1. Time–Frequency Power Topographic Regions Induced by IMT

**Figure 4:**
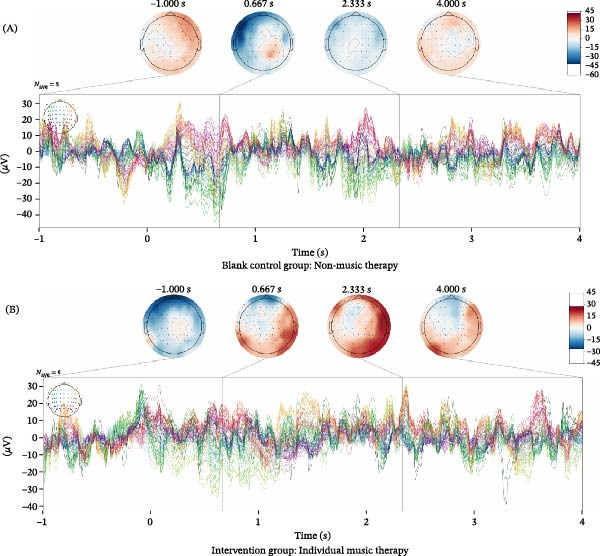
Topographic maps of EEG spectral power for the blank control group (A) and the individual music therapy (IMT) group (B), recorded with 128 channels. Cool colors indicate lower power; warm colors indicate higher power. Power values are displayed in relative units (software analysis by Neuracle‐Seal). No inferential statistics were applied to these topographic maps.

In the blank control group, left frontal–temporal–parietal regions showed decreased spectral power (cool colors, Figure 4). In the IMT group, right frontal‐temporal‐parietal regions showed increased spectral power (warm colors, Figure 4). These topographic patterns are presented descriptively; no inferential statistics were applied to the topographic maps. (Software analysis by Neuracle‐Seal).

**Table 3:** Detailed sub‐item analysis (ANCOVA adjusted).

Sub‐item dimensions	IMT (T0 → T1)	Control (T0 → T1)	Adjusted beta	*p*‐Value
(1) Sight	0.45 → 2.45	0.48 → 1.48	−0.99	<0.001
(2) Hearing	0.73 → 3.73	0.57 → 2.57	−1.00	<0.001
(3) Stimulus to musical sensations	0.64 → 3.64	0.62 → 1.62	−2.00	<0.001
(4) Verbal commands	0.55 → 0.95	0.57 → 1.33	0.36	0.071
(5) State of consciousness	0.55 → 1.32	0.57 → 1.14	−0.19	0.255
(6) Behavioral reaction to music	1.41 → 1.95	0.57 → 1.57	0.06	0.587
(7) Reaction to music	1.45 → 1.95	1.24 → 1.48	−0.41	0.001
(8) Vocalization	0.82 → 2.36	0.62 → 0.90	−1.32	<0.001
(9) Nonverbal communication	1.14 → 2.18	1.14 → 1.95	−0.23	0.016
(10) Decision making	0.52 → 0.91	0.26→ 0.45	−0.49	0.019
(11) Mobility	0.57 → 1.18	0.57 → 0.81	−0.38	0.020
(12) Attention to tasks	1.09 → 2.09	1.05 → 2.05	−0.002	0.982
(13) Directed actions	0.91 → 2.45	0.48 → 1.48	−0.73	<0.001
(14) Emotional reactions	2.27 → 4.27	2.29 → 3.24	−1.05	<0.001

*Note:* Adjusted beta represents the group effect (control relative to IMT) from ANCOVA models with T0 score as a covariate.

#### 3.2.2. The Time Series Analysis Results of ERSP and Inter‐Trial Phase (ITP) Locking Value

**Figure 5:**
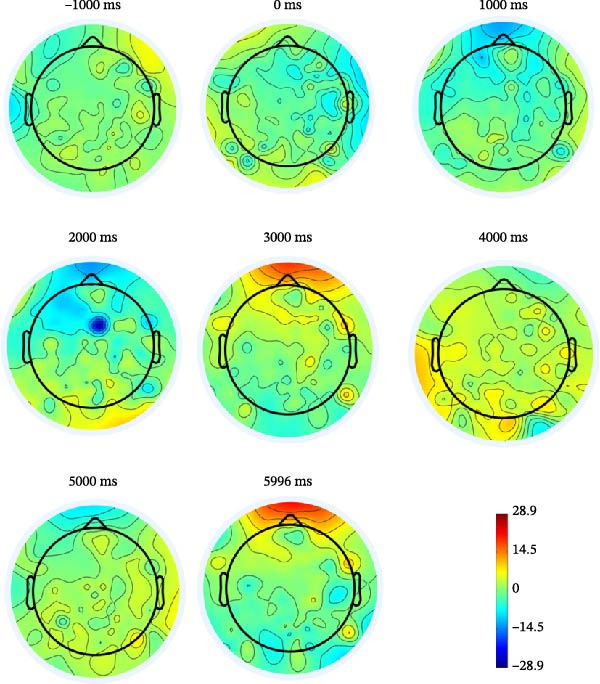
Topographic map of EEG spectral power at 3000 ms after stimulus onset (music onset) for the IMT group. Warm colors indicate higher power; cool colors indicate lower power. This map is presented descriptively; the terms “enhanced/active” or “reduced/suppressed” are not intended to imply mechanistic interpretations (software analysis by Neuracle‐Seal).

In the blank control group, spectral power changes were observed within the 5–10 Hz range between 200 and 600 ms, as well as within the 10–50 Hz range (Figure 2). In the IMT group, power changes were most prominent around 200 ms, primarily within the 5–10 Hz range (theta and alpha bands). The duration of power changes in the IMT group extended from approximately 200 to 1400 ms (Figure 2). The blank control group showed power changes across a broader frequency range but with shorter duration, whereas the IMT group showed more concentrated power within the 5–8 Hz range (Figure 2) and at the 3000 ms time point (Figure 5), with a slower decay after 6000 ms (Figures 2 and 5). All time–frequency results are presented for descriptive purposes; no statistical comparisons were performed across groups for the full time–frequency plane (Software analysis by Neuracle‐Seal).

### 3.3. The Brain Region Activation Results of the EEG 3D Image

**Figure 6:**
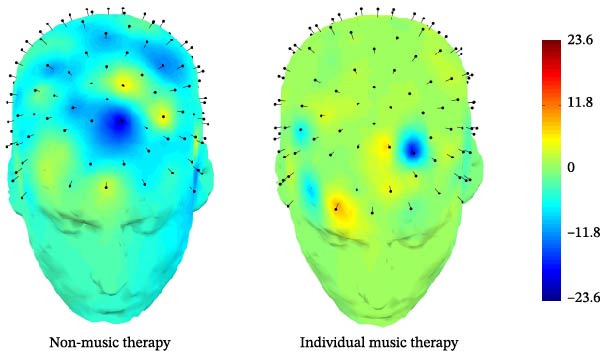
3D topographic maps of EEG spectral power for the IMT group between 200 and 1400 ms. Warm colors indicate higher power; cool colors indicate lower power. These 3D images are based on EEGLAB’s default source estimation and should be interpreted with caution given the limited spatial accuracy of EEG source localization, especially in patients with brain lesions. The term “significant” is not used because inferential statistics were not applied to these maps.

**Figure 7:**
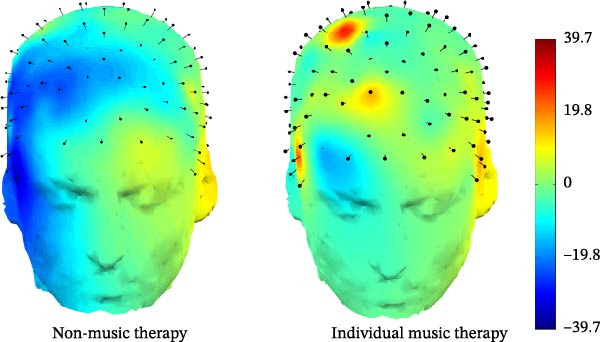
3D topographic maps of EEG spectral power for the IMT group between 3000 and 6000 ms, and after 6000 ms. Warm colors indicate higher power; cool colors indicate lower power. As in Figure 6, these images are descriptive and derived from EEGLAB’s default source estimation; they do not represent statistically tested “activation” and should not be overinterpreted (software analysis by EEGLAB). For all topographic and 3D images, spectral power is displayed in relative units. The maps are presented for descriptive purposes only; no statistical inference was performed on the spatial distribution of power unless otherwise stated.

In the 3D EEG images, the IMT group showed higher brain activation areas, mainly concentrated in the lateral prefrontal cortex, precentral gyrus, and central sulcus on the right side of the brain. Additionally, there was marked inhibition in the precentral gyrus of the left brain. In the blank control group, the patients’ brains remained in a resting state, with most areas of the frontal, parietal, and occipital lobes showing minimal activity, suggesting inhibition (As is illustrated in Figures 6 and 7, software analysis by EEGLAB). Music stimulation leads to MCS.

## 4. Discussion

Music holds personal meaning—especially songs that were meaningful to patients before their illness, such as their favorite or most emotionally significant pieces. Using these insights to personalize music therapy (i.e., IMT) can spark stronger and more distinct responses in the brain and in behavior compared with music therapy using music unfamiliar to the patient. Responses to IMT may be observable, such as opening of the eyes, turning of the head, or even tears, as measured by the MATADOC scale, or they may be subtle, such as changes in brain activity that are detectable through EEG. At the heart of IMT is the belief that music is closely tied to a person’s past, connected to cherished memories, identity, and deep emotions, and can help with awareness and engagement. This could include childhood lullabies, songs from important life moments, such as weddings or graduations, or anthems linked to military services. The goal of IMT is to use these deeply personal musical experiences to activate key brain networks and support improved connections within the brain, offering a pathway toward reconnection and healing.

### 4.1. Validity of MATADOC Behavioral Assessment

This study investigated the use of IMT for patients in an MCS, with positive results observed in the post‐intervention MATADOC assessments. The patients showed meaningful progress in auditory perception and attention, emotional responsiveness, vocalization, and overall behavioral engagement with music. During the IMT sessions, individuals in the experimental group began to respond more clearly to familiar sounds, for instance, turning their heads toward the music source, indicating an improved ability to locate sounds. Many of the participants demonstrated longer periods of focused listening and more consistent reactions when listening to the same music multiple times. These positive changes suggest that IMT supports auditory attention development.

One key observation was found during follow‐up evaluations. Some patients who previously did not respond to any sounds began to naturally turn their gaze toward the music therapist during live performances. This small but significant change reflected improvements in behavioral responses, contributing to higher MATADOC scores.

IMT also had a remarkable impact in helping patients reconnect emotionally. When they heard personally meaningful music, the music appeared to gently awaken deep emotional centers in the brain, leading to visible signs of feeling. Some patients shed tears during reflective or sentimental songs. Other participants showed sudden eye movements or shifts in breathing, such as sighing, in response to uplifting melodies. These responses are important markers within the “affective response” section of the MATADOC, representing moments of emotional awareness.

IMT can create novel nonverbal ways for patients to express themselves. The music therapists noticed that some individuals approaching wakefulness began to show preferences through their behavior. For example, calming and alert attention often signaled the enjoyment of familiar music, while drowsiness suggested less connection with unfamiliar pieces. Earlier research has shown that rhythmically engaging music can help increase alertness, which concurred with our findings. In our study, faster tempo music (e.g., 120 bpm) was linked to longer periods of eye‐opening and greater overall alertness, pointing to a more stable and responsive state of awareness.

Overall, these results highlight that IMT can lead to significantly greater improvements in MATADOC scores than general auditory stimuli. This reinforces the role of personalization in effective music therapy. Music is tied to a person’s life story, memories, and emotions. These not only convey familiarity but can also be used to activate key brain networks related to self‐awareness, emotion, and memory, offering a gentle, human‐centered path toward reconnection and healing.

### 4.2. EEG During IMT in Patients With DoC

This study demonstrated that IMT can support brain activation in patients with MCS, particularly those in an MCS. One of the most meaningful findings suggests that personalized music can help engage the right hemisphere of the brain, which is deeply involved in processing melodies, rhythms, and emotions [25]. Music naturally activates the widespread neural networks related to hearing, memory, feeling, and attention, which, in turn, offers a unique pathway to stimulate areas that may still be functionally preserved, even when awareness is limited.

Patients in the IMT group listened to music that was personally meaningful to them, such as songs tied to their life experiences, cultural backgrounds, or emotional memories. EEG recordings during IMT revealed that this tailored approach led to stronger theta–gamma PAC in the right hemisphere (Figure 4), especially in the fronto‐temporo‐parietal regions. These changes emerged shortly after the music began and reflected increased neural activity linked to conscious processing. Notably, this pattern of brain response was closely associated with improvements in clinical behavior. For example, one patient with a history of childhood performances showed strong brain activation over the right fronto‐temporo‐occipital areas when hearing familiar songs such as “Listening to Mother’s Past Stories” and “I Love the Blue Sky of My Motherland” (Figure 4). Such self‐referential music appeared to awaken the DMN, a key system involved in self‐awareness and memory, which, in turn, helped coordinate broader brain connectivity in the patient. Whole‐brain engagement supports sustained arousal and environmental awareness, both of which are essential for recovery.

Additionally, music can help the brain better detect and respond to meaningful stimuli by activating the salience network, which is an important bridge between internal states and external inputs [26]. This may explain why patients responded more fully and consistently during the IMT sessions.

Time–frequency analyses using the ERSP and ITP showed clear differences between the two groups. In the control group, everyday sounds, such as conversations, triggered brief responses across multiple brainwave bands, but these faded quickly after the sound ended. In contrast, patients who experienced live musical performances responded rapidly—within just 200 ms—and continued to show lingering brain activity in *θ*‐ and *α*‐waves even after the music stopped. This prolonged effect suggests that music, especially live singing connected to personal and cultural memories, resonates deeply with preserved neural rhythms in these patients, creating lasting neural impressions that support ongoing awareness.

3D brain imaging further confirmed that music directly activates critical regions, such as the right lateral prefrontal cortex, precentral gyrus, and central sulcus, which are responsible for conscious control and sensory integration. IMT not only sparked positive emotions and memories but also strengthened connections within the right hemisphere and limbic system. Brain maps from the experimental group suggest that patients with MCS may have higher levels of hidden awareness than what can be observed through behavior alone, a phenomenon known as cognitive‐motor dissociation [27]. Under music stimulation, their resting‐state brain activity became more dynamic, showing richer and more integrated patterns, particularly in the α frequency band.

Over time, patients who received IMT showed greater reorganization and recovery of the neural networks than those in the control group. Following IMT, the patients’ brains demonstrated improved functional connectivity, both within the right hemisphere and across distant regions, supporting natural compensation and healing processes. While the control group showed some normal posterior α activity and initial responses to speech, these effects were short‐lived. In contrast, the IMT group exhibited responses characterized by “rapid onset, high frequency, and sustained duration,” a promising signature of deeper engagement.

All participants in the IMT group had regular music sessions with certified music therapists over a 12‐week study period. Each patient’s playlist was customized based on the patient’s pre‐illness life, including the cultural context, education, and personal taste. This deep level of personalization is likely critical to stimulating the core networks of the brain, especially the frontotemporoparietal systems, which are often affected by DoC. Importantly, although personalized music tends to activate the right hemisphere during emotional and melodic processing, it also encourages balanced communication between the two hemispheres via the corpus callosum. Synchronization across the whole brain is a crucial step toward restoring conscious awareness.

EEG has come a long way from its early use as a basic tool for assessing brain function to becoming a sophisticated, multidimensional system that offers deep insights into the experiences of patients with MCS. EEG has evolved to be much more than just a support for diagnosis and prediction of outcomes. EEG serves as a meaningful bridge, helping us detect signs of hidden awareness and facilitating a pathway to connect with individuals who cannot communicate in traditional ways. At the same time, EEG provides an objective way to track changes over time, making it a valuable tool for monitoring how patients respond to treatment. In managing MCS, combining behavioral assessments (such as CRS‐R and MATADOC) with neurophysiological methods, such as EEG, and advanced imaging techniques, such as fMRI, is increasingly recognized as the most reliable approach—a “gold standard”—for achieving accurate diagnosis and personalized prognosis.

### 4.3. Limitations

This study has limitations. Forty‐three patients completed the 12‐week trial. The small sample size limited the power of the study. An additional blank control group to observe self‐healing may have enhanced the study. In the future, studies with a larger sample size to validate therapeutic outcomes more precisely are warranted. The MATADOC has not been formally validated in Chinese MCS patients, which is acknowledged as a limitation.

### 4.4. Implications for Clinical Practice

Traditionally, clinicians would use different therapies to treat patients with MCS but overlook the function of music stimulation. In recent years, evidence suggests that music plays an important role in the recovery of patients with MCS. Although music therapy has been proposed and used worldwide for over 80 years, it is often used in psychotherapy, pediatrics, and other alternative therapies. Given that professionals with a musical background, that is, music therapists, have a more professional understanding of music or songs and the operability of the musical instrument, the IMT performed by the music therapist can provide multiple auditory stimulations to the patients to activate more potential brain networks and better restore arousal ability. In this study, we confirmed the positive effects of IMT, performed by a music therapist, on 22 patients with MCS. All participants in the intervention group were more active in every aspect of the MATADOC assessment than those in the control group, suggesting that IMT may be an effective approach for the rehabilitation of patients with MCS. Music therapists with professional backgrounds provide multiple auditory stimuli with instrumental accompaniment and customized musical biographies during the treatment process, which are necessary for the implementation of IMT.

## 5. Conclusions

IMT performed by professional music therapists resulted in a more obvious effect on the consciousness of patients with MCS. In the future, studies conducted collaboratively by clinicians and professional music therapists to improve the clinical efficacy of IMT are warranted.

## Author Contributions

Xiaoying Zhang was responsible for writing the manuscript, study design, patient allocation, protocol development, results description, statistical chart drawing, clinical analysis, and discussion. Yunlei Wang and Zuliyaer Talifu were responsible for the data analysis. Qingqing Feng and Jiayi Gu were responsible for music therapy and behavioral assessment. Xiaobing Li provided consultation.

## Funding

This work was supported by the Capital Funds for Health Improvement and Research (Grant CFH 2024‐2‐6011) and the National Natural Science Foundation of China (NSFC) (Grant T2341003).

## Disclosure

No adverse events related to the music therapy intervention or EEG recording were observed or reported during the entire trial period. Vital signs remained stable in all participants before, during, and after each session. The writing and editing of the article were performed in accordance with the CONsolidated Standards Of Reporting Trials (CONSORT) Statement. The statistical methods of this study were reviewed by the epidemiologist of Capital Medical University and Chinese Academy of Medical Sciences/Peking Union Medical College, China.

## Consent

The authors certify that they have obtained the consent forms from patients. In the form, patients have given their consent for their images and other clinical information to be reported in the journal. The patients understand that their names and initials will not be published.

## Conflicts of Interest

The authors declare no conflicts of interest.

## Data Availability

The data that support the findings of this study are available from the corresponding author upon reasonable request. The individual deidentified participant data (including data dictionaries) will be shared, along with related documents such as the study protocol and statistical analysis plan. The data will become available in the future within 5 years. Research colleagues can access the data through the China Clinical Trials Registry, and the Resman clinical trials public administration platform.
